# Metagenomic Analysis of Marigold: Mixed Infection Including Two New Viruses

**DOI:** 10.3390/v13071254

**Published:** 2021-06-28

**Authors:** Hang Yin, Zheng Dong, Xulong Wang, Shuhao Lu, Fei Xia, Annihaer Abuduwaili, Yang Bi, Yongqiang Li

**Affiliations:** 1 College of Bioscience and Resource Environment, Beijing University of Agriculture, Beijing 102206, China; bank0919@163.com (H.Y.); dz199838@sina.com (Z.D.); wxl17812090398@163.com (X.W.); 13629840525@163.com (S.L.); anddy3838@163.com (A.A.); biyang0620@126.com (Y.B.); 2 Key Laboratory for Northern Urban Agriculture of Ministry of Agriculture and Rural Affairs, Department of Plant Protection, Beijing University of Agriculture, Beijing 102206, China; 3 Beijing Institute of Landscape Architecture, Beijing 100102, China; xiafeirena@163.com

**Keywords:** marigold, deep sequencing, mixed infection, new virus

## Abstract

Marigold plants with symptoms of mosaic, crinkle, leaf curl and necrosis were observed and small RNA and ribo-depleted total RNA deep sequencing were conducted to identify the associated viruses. Broad bean wilt virus 2, cucumber mosaic virus, turnip mosaic virus, a new potyvirus tentatively named marigold mosaic virus (MMV) and a new partitivirus named as marigold cryptic virus (MCV) were finally identified. Complete genome sequence analysis showed MMV was 9811 nt in length, encoding a large polyprotein with highest aa sequence identity (57%) with the putative potyvirus polygonatumkingianum virus 1. Phylogenetic analysis with the definite potyviruses based on the polyprotein sequence showed MMV clustered closest to plum pox virus. The complete genome of MCV comprised of dsRNA1 (1583 bp) and dsRNA2 (1459 bp), encoding the RNA-dependent RNA polymerase (RdRp), and coat protein (CP), respectively. MCV RdRp shared the highest (75.7%) aa sequence identity with the unclassified partitivirus ambrosia cryptic virus 2, and 59.0%, 57.1%, 56.1%, 54.5% and 33.7% with the corresponding region of the definite delta-partitiviruses, pepper cryptic virus 2, beet cryptic virus 3, beet cryptic virus 2, pepper cryptic virus 1 and fig cryptic virus, respectively. Phylogenetic analysis based on the RdRp aa sequence showed MCV clustered into the delta-partitivirus group. These findings enriched our knowledge of viruses infecting marigold, but the association of the observed symptom and the identified viruses and the biological characterization of the new viruses should be further investigated.

## 1. Introduction

Viral diseases are a major threat to sustainable and productive agriculture worldwide, resulting in economic losses in grain, fruit, vegetable and ornamental plants [1]. Plant viruses have evolved rapidly due to the high mutation rate and frequent recombination, which challenged the traditional techniques for virus diagnosis. The advent of high-throughput sequencing (HTS) technologies, also known as next-generation sequencing, has revolutionized plant virus diagnosis [2,3]. HTS does not require any viral sequence knowledge and can sequence millions or billions of DNA molecules in parallel followed by sequence assembly and annotation, enabling the *de novo* detection of all viruses present in a plant, including those still unknown [4]. Thus, it is effective for the discovery of unknown etiological agents or in quarantine analysis of plant material trades, especially in the case of long known plant diseases of unknown etiology [5,6,7,8].

Ornamental plants constitute a potential source of unknown viruses and viroids that not only affect ornamental plants, but also could have a major impact on agriculture as alternate hosts [9]. Next generation sequencing is an effective means for screening the presence of potentially important pathogens in ornamental plants, which may lead to more severe epidemiological risks due to virus and viroid diseases of ornamentals in the globalization of the ornamental plant industry. Marigold (*Tagetes* *erecta* L.), an annual herbaceous plant from the genus *Tagetes* and family Asteraceae, is native to Mexico and cultivated commercially as a popular garden ornamental for its wide spectrum flowers with attractive color, shape and size [10]. It is also widely cultivated as a dye plant and source of marigold meal [11]. In addition, it has a wide range of medicinal uses for antimicrobial, antiseptic, wound and ulcer healing, and hypotensive properties, due to its rich carotenoids, essential oils and flavonoids [12,13]. The flavonoids, amides, and phenols from *T. erecta* root have been reported to have insecticidal and nematicidal effects and sometimes it is planted as an intercrop with cowpea for nematode pest control [14]. Inspite of the insecticidal, fungicidal, bactericidal, and larvicidal properties of marigold it is also affected by various pathogenic microorganisms such as fungi, viruses and bacteria that cause diseases and damage to the plant which result in yield loss. Up to now, ageratum enation virus [15], cucumber mosaic virus [16], papaya ringspot virus [17], potato virus Y [18], potato yellow dwarf virus [19], potato yellow vein virus [20], tobacco streak virus [21,22], tomato spotted wilt virus [23,24,25,26] and tomato yellow ring virus [27] have been reported in marigold plants, causing the symptoms of severe stunting, yellowing, mosaic, leaf curl, crinkle and necrosis.

In August 2018, marigold plants with the symptom of mosaic, crinkle, leaf curl and necrosis were observed in Huairou, Beijing. To characterize the viruses associated with this disease, small RNA deep sequencing and ribo-depleted total RNA were conducted. Sequence analyses revealed mixed virus infections including three known viruses: CMV, broad bean wilt virus-2 (BBWV-2), turnip mosaic virus (TuMV), and two novel viruses tentatively named marigold mosaic virus (MMV) in *Potyvirus* and marigold cryptic virus (MCV) in *Deltapartitivirus*.

## 2. Materials and Methods

### 2.1. Plant Materials

**Figure 1:**
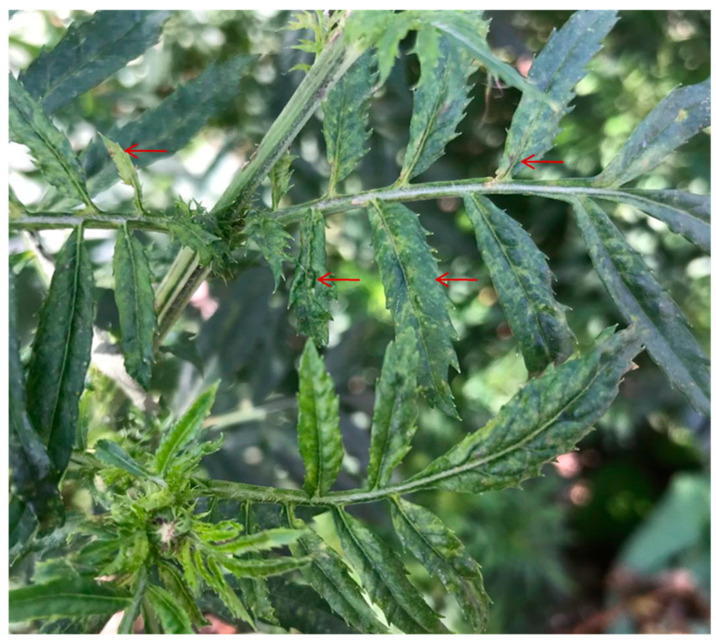
Mosaic, leaf curl, necrosis and crinkle symptoms observed in marigold.

Marigold plants with the symptoms of mosaic, crinkle, leaf curl and necrosis were observed in Huairou, Beijing in August, 2018 (Figure 1). The leaves from a symptomatic plant were collected and the total RNA was extracted from the samples with TRIzol reagent (Invitrogen, Carlsbad, CA, USA) according to the manufacturer′s instructions. Then the extracted RNA was quantified and assessed for quality using a Nanodrop ND-1000 (Nanodrop Technologies, Wilmington, DE, USA) and integrity using Bio-Analyzer 2100 (Agilent Technologies, Waldbronn, Germany). Total RNA with high quality was then used for the following deep sequencing.

### 2.2. Library Construction, Deep Sequencing and Bioinformatic Analysis

A small RNA library was constructed with the TruSeqSmall RNA Sample Prep Kit (Illumina, San Diego, CA, USA) following the manufacturer’s instructions. Generally, small RNAs of 18–28 nt were isolated from the total RNA with 15% polyacrylamide gel for 5′ and 3′ adaptors ligation. The final ligation products were purified and reverse-transcribed followed by PCR for sequencing library construction. Deep sequencing was performed according to the manufacturer′s instruction using the Illumina Hiseq2500 sequencing platform. Raw data was processed to trim the adaptors and remove the sequences smaller than 16 nucleotides (nt) or longer than 30 nt, low-quality tags, polyA or N tags with an in-house perl script. The resulting clean reads were assembled into contigs with Velvet which were further annotated by BLASTn and BLASTx searches against the GenBank database. For ribo-depleted total RNA sequencing, the ribosomal RNA was removed from the total RNA with the Ribo-ZeroTM rRNA Removal Kit (Epicentre, Madison, WI, USA) and the cDNA library was then prepared with the TruSeq RNA Sample Prep Kit v2 (Illumina, CA, USA) according to the manufacturer′s protocol, which was deep sequenced on the Illumina Hiseq2500 platform. The raw reads were quality trimmed with SOAPnuke [28] and further *de novo* assembled into contigs with Trinity [29] and annotated by BLASTn and BLASTx searches against the GenBank database. To characterize the virus derived small interfering RNA (vsiRNA), the sequences of clean reads that were identical or complementary to candidate virus genomic sequence were recognized as vsiRNAs for further analyses with Bowtie software.

### 2.3. Genome Sequence Reconstruction of Candidate New Viruses

The complete genome sequence was RT-PCR amplified with sufficiently overlapped primer pairs and RACE for both 5′ and 3′ terminal sequences (Table S1). The RT reactions were performed using the PrimeScript II 1st Strand cDNA Synthesis Kit (Takara, Dalian, China) and PCR was performed in a 25 μL reaction volume containing 1μL cDNA, 1× LA *Taq* buffer, 10 mM dNTPs, 1U LA *Taq* polymerase, 100 mM of each primer (Table S1) and sterile double distilled water to a final volume. The mixture was incubated at 94 °C for 5 min, followed by 30 cycles of 94 °C for 30 s, 54 °C for 30 s and 72 °C for 2 min, and an additional elongation step at 72 °C for 10 min. The amplified products were purified and ligated into pMD19-T vector, and then transformed into *E.coli* DH5α competent cells. At least five independent clones of each fragment were sequenced. RACE PCR was conducted with SMARTer RACE 5′/3′ kit following the manufacturer’s instructions.

### 2.4. Sequence Analysis

The coding capacity of candidate virus was identified using the NCBI ORFfinder (https://www.ncbi.nlm.nih.gov/orffinder, 21 September 2020). Conserved domains of the putative gene products were searched using the conserved domain tool (http://www_ncbi.xilesou.top/Structure/cdd/wrpsb.cgi, 21 September 2020). For phylogenetic analyses, the virus genome sequences were downloaded from GenBank database and phylogenetic tree was constructed with the aligned sequences with Clustal W [30] using the Neighbor-Joining (NJ) methods implemented in the MEGA6.06 program [31] with the best-fit models recommended by a model test in this software.

### 2.5. Field Detection of Candidate Viruses

Leaf samples from 24 marigold plants with the symptom of mosaic, crinkle, and/or leaf curl (15 from Huairou, 9 from Yanqing) and 10 asymptomatic plants (6 from Huairou and 4 from Yanqing) were collected and total RNA was extracted from each sample using the TRIzol reagent. RT-PCR was conducted as described above (primers are listed in Table S1) and the resulting amplicons were confirmed by electrophoresis on a 1.0% agarose gel, stained with ethidium bromide and visualized under UV light.

## 3. Results

### 3.1. Deep Sequencing Analyses

In the sRNA sequencing library, a total of 8,911,917 clean reads with the dominant size of 21- (25.6%), 22- (10.0%) and 24 nt (32.4%) were obtained. After *de novo* assemble with Velvet 9,495 contigs with sizes ranging between 33 and 791 nt were gained, which were further annotated by BLAST. 263 contigs were identified to be virus originated and related to BBWV-2 (84 contigs of 8,577nt with 97.8% genome coverage), CMV (34 contigs of 6,821 nt with 77.4% genome coverage), poly-gonatumkingianum virus 1 (PKV1, 27 contigs of 8247nt with 88.1% genome coverage) and ambrosia cryptic virus 2 dsRNA1 (AmCV2, 6 contigs of 454 bp with 28.2% coverage of dsRNA1) (Table 1). To further characterize the genome sequence of the identified virus related to AmCV2 dsRNA2, ribo-depleted RNA sequencing was conducted. In the ribo-depleted RNA sequencing 224,941,546 clean reads were gained and 154,372 contigs were assembled. Further BLAST analysis identified five contigs with the assembled length of 9465 nt related to BBWV 2 (94% and 96% nt sequence identity with the RNA1 and RNA2, respectively), four contigs with the length of 8634 nt related to CMV (99% nt sequence identity with RNA1-3 respectively), one contig of 9811 nt (97% nt sequence identity with TuMV), one contig of 9798 nt with 57.2% nt sequence identity of PKV1 and two contigs of 1583 bp and 1459 bp with 72.6% and 38.9% nt sequence identity with the dsRNA1 and dsRNA2 of AmCV2, respectively (Table 1). The presence of these viruses was all confirmed by RT-PCR with the corresponding primer pairs in Table S1.

### 3.2. Three Known Viruses in Marigold-CMV, BBWV-2 and TuMV

The genomic RNA of TuMV from marigold (TuMV-marigold) was 9811 nt in length excluding the 3′ poly(A) tail (MW556590). BLAST analyses showed TuMV-marigold shared 97.6% nt and 99.0% aa sequence identity, the highest with the isolate HRD from *Raphanus sativus*(AB093627.1). The genomic RNA encoded a large polyprotein which was further cleaved into ten mature proteins and these sequences encompass regions encoding the P1, HC-Pro, P3, 6K1, CI, 6K2, VPg,NIa-Pro,NIb and CP genes with 1086, 1374, 1065, 156, 1932, 159, 576, 729, 1551 and 864 nt, respectively. All the conserved motifs in potyviruses were present in TuMV-marigold. For the CMV infecting marigold, the three genomic RNAs were 3390-, 3040- and 2204 nt in length for RNA1, RNA2 and RNA3, respectively (MW556587-MW556589 for RNA1-3). RNA1 encoded the 1a protein of 933 aa with an estimated molecular weight of 111.2 kDa; RNA2 encoded the 830 aa long 2a protein of 94.3 kDa known as RNA-dependent RNA polymerase and the 100 aa long 2b protein of 11.6 kDa, a well-known gene silencing suppressor; RNA3 encoded the 279 aa long 3a protein of 30.2 kDa and the 218 aa CP of 24.2 kDa. BLAST analysis showed the three RNAs and their encoded proteins shared highest nt and aa sequence identities with the isolate SL in the subgroup II. The bipartite genome of BBWV-2 was 5926 nt for RNA1 and 3583 nt for RNA2 (MW556591 for RNA1 and MW556592 for RNA2), each encoding a polyprotein. RNA1 encoded the polyprotein 1 of 1870 aa with a calculated molecular weight of 209.7 kDa, and shared highest aa sequence identity (98.7%) with the isolate PatMMV and 89.2%–98.2% sequence identities with other reported isolates. Conserved cleavage sites Q/D, Q/S, Q/S, Q/G were identified in polyprotein 1 for the mature proteins Co-Pro, helicase, Vpg, proteinase, and RdRp. All the conserved motifs in these mature proteins were also identified. RNA2 encoded the polyprotein 2 of 1064 aa (118.6 kDa) and shared highest aa sequence identity (97.9%) with the isolate Gyp and 87.8%–96.6% sequence identities with other reported isolates. Polyprotein 2 was cleaved into the MP, LCP and SCP at the sites of Q/G and Q/A.

### 3.3. A potential New Member in Deltapartitivirus-Marigold Cryptic Virus

**Figure 2:**
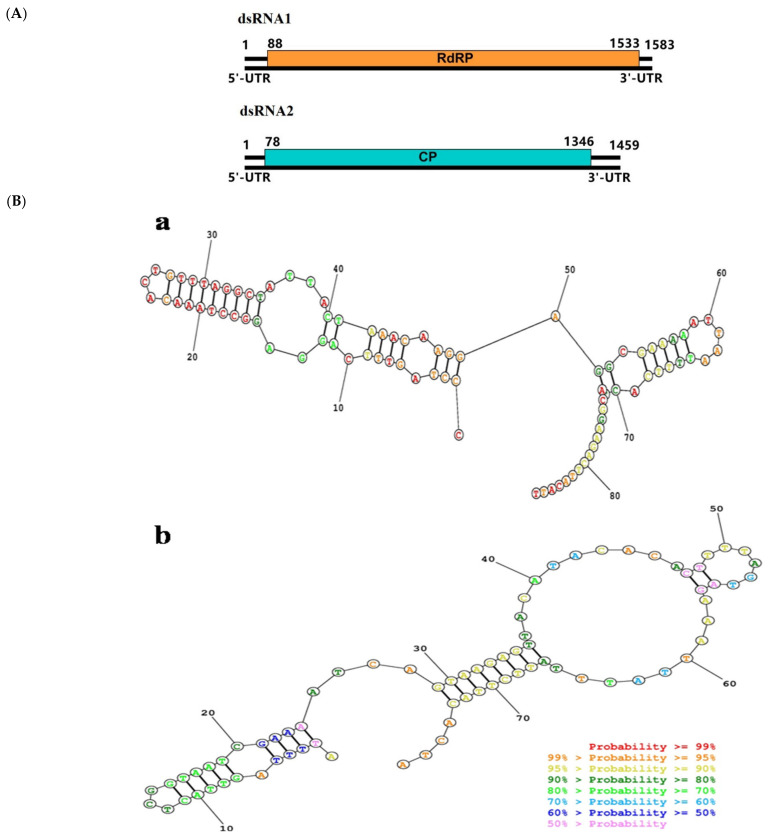

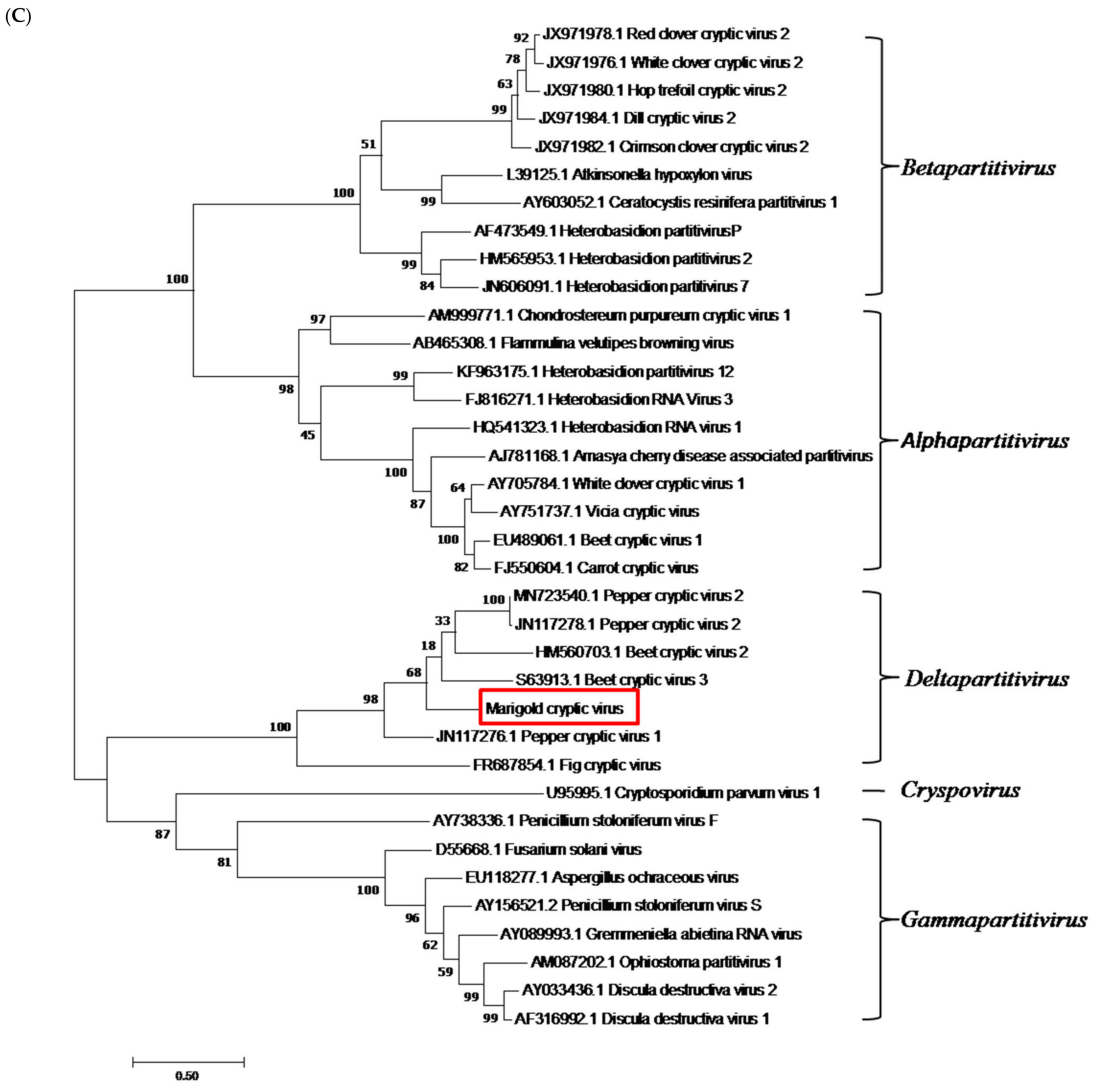
Genome structure of marigold cryptic virus (MCV) and its phylogenetic analyses. (**A**): Diagram showing genome organization of MCV and open reading frames (ORFs) were indicated as boxes on each segment and the ORF positions were also indicated. (**B**): The secondary structure formed by the 5′-untranslated regions (5′UTR) of MCV dsRNA-1 (**a**) and dsRNA-2 (**b**). (**C**): Neighbor-joining tree based on the aa sequence of RdRp of classified members of the family *Partitiviridae* with 1000 bootstrap replicates. Bootstrap values were given by numbers at the relevant nodes in the topology.

The complete genomic sequences of MCV consisted of two dsRNAs, dsRNA1 of 1,583 bp and dsRNA2 of 1459 bp (MW546937 for dsRNA1 and MW546938 for dsRNA2), within the length range of delta-partitiviruses. Similar to those of other known two-segmented partitiviruses, the genome organization of MCV contained two ORFs, one on each segment (Figure 2A). The ORF1 (bp 88–1533) encoded a 481 aa protein of 54.5 kDa with conserved RdRp motif (pfam00680) at aa position 225–402. The conserved motifs, known to be related to virus replication [32], were also present in MCV RdRp, including the motif I (_129_SSSAGYGY_136_), II (_179_PDVGYTRTQL_18__8_), III (_195_TKVRGVW_201_), IV (_257_DWSQFDSTVS_266_), V (_317_GIPSGSYFTTLIGSIVNRLRI_337_), VI (_354_VLGDDSLI_361_), VII (_398_TFLGR_402_), and VIII (_417_LRLLILPEYP_426_). The ORF 2 (bp 78–1346) encoded the CP of 422 aa with an estimated molecular weight of 48.7 kDa. The sequence 5′-AGAATTTTC-3′ was found highly conserved in the delta-parititiviruses 5′-terminus of both the dsRNA-1 and dsRNA-2, but in MCV this sequence was not found. In silico analysis of the secondary structures of the 5′UTRs of dsRNA-1 and dsRNA-2 with Mathews RNA structure (http://rna.urmc.rochester.edu/RNAstructureWeb/Servers/Predict1/Predict1.html, 27 January 2021) identified stable stem-loop structures in these regions, which was likely to be involved in RNA packaging and/or replication [33] (Figure 2B). In the family *Partitiviridae*, the genus demarcation criteria was based on the characteristic hosts within each genus (plants for genera *Alphapartititivirus*, *Betapartitivirus* and *Deltapartitivirus*), genome segment and protein lengths, RdRp amino acid sequence identity (<24%), and phylogenetic grouping of RdRp and species demarcation in each genus was ≤90% aa sequence identity in the RdRp, and ≤80% aa sequence identity in the CP. Comparison of RdRp aa sequence showed MCV shared the highest (75.7%) sequence identity with unclassified partitivirus AmCV2, and 59.0%, 57.1%, 56.1%, 54.5% and 33.7% with the corresponding region of the definite members in the genus *Deltapartitivirus*, pepper cryptic virus 2 (PepCV2), beet cryptic virus 3 (BCV3), beet cryptic virus 2 (BCV2), pepper cryptic virus 1 (PepCV1) and fig cryptic virus (FiCV), respectively. Moreover, the MCV RdRp shared low aa sequence identities (13.3%–15.3%) with the members in *Alphapartitivirus*. Multiple aa sequence alignment of MaCV CP showed it shared the highest (43.1%) sequence identity with the dactylorhiza cryptic virus 2, an unclassified partitivirus. Further phylogenetic analysis based on RdRp aa sequence showed all the partitiviruses clustered into five phylogenetic clades, and our newly identified virus MCV formed a well separated branch together with FiCV, PepCV1, PepCV2, BCV2, BCV3 (Figure 2C). Based on the above results, the virus MCV in this study should be considered as a new species within the genus *Deltapartitivirus*.

### 3.4. Characterization of a New Potyvirus-Marigold Mosaic Virus

**Figure 3:**
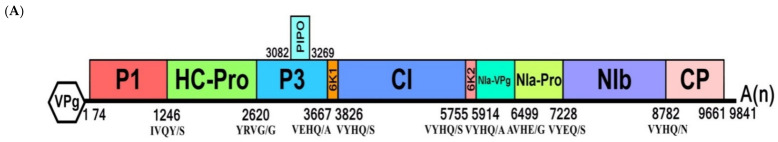

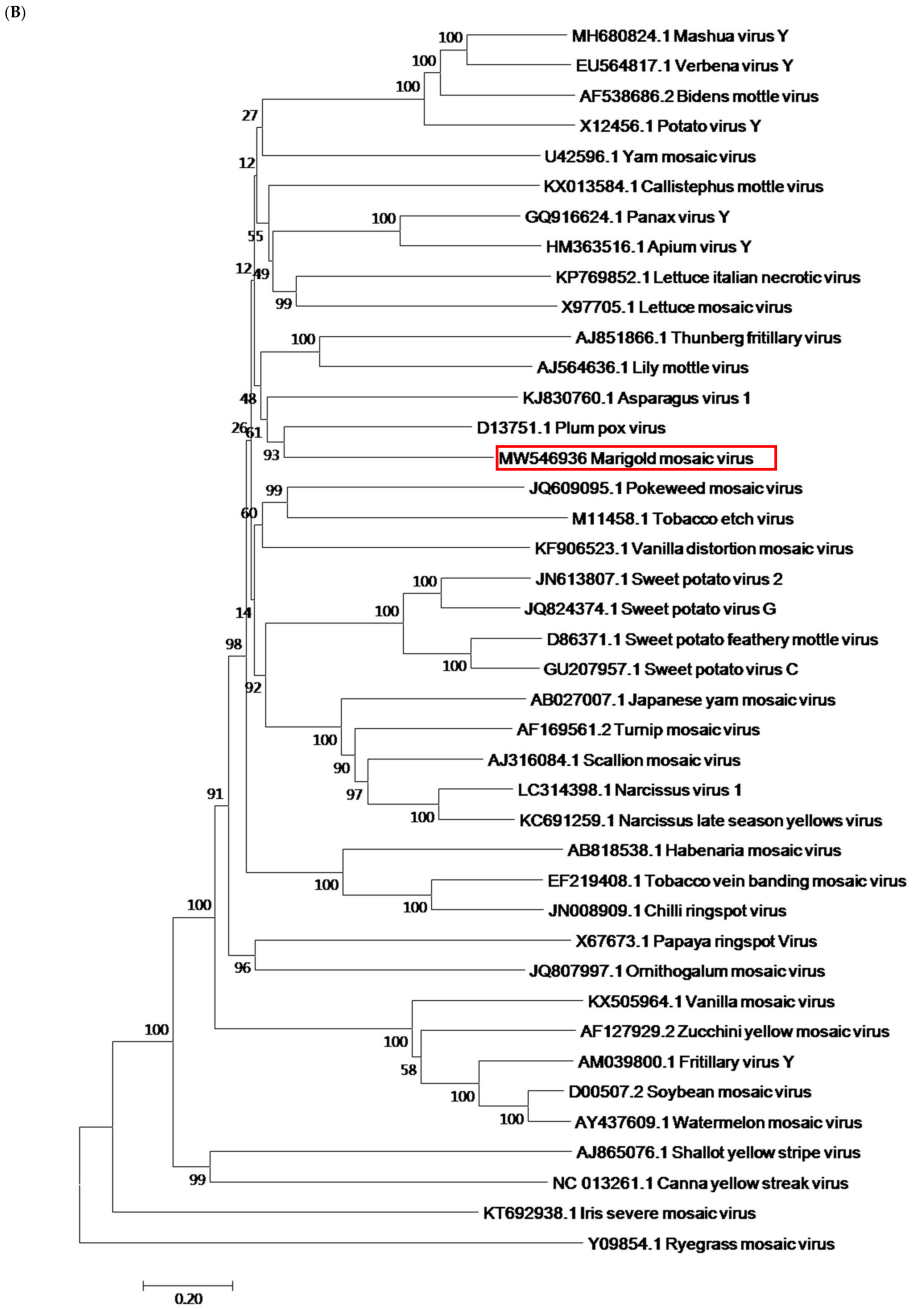
Genome structure of marigold mosaic virus (MMV) and its phylogenetic analysis. (**A**): Schematic representation of the genomic organization of MMV. The position of the mature protein and the proteinase cleavage sites were listed. (**B**): Neighbor-joining tree based on the polyprotein aa sequence of the members in the family *Potyviridae* with 1000 bootstrap replicates. Bootstrap values were given by numbers at the relevant nodes in the topology. Ryegrass mosaic virus was used as outgroup.

The genomic RNA of MMV was 9841nt in length (MW546936), excluding the poly(A) tail at the 3′ end and ORFfinder analysis identified an ORF encoding a polyprotein in the genome with the 5′ and 3′ untranslated regions of 73 and 178nt, respectively (Figure 3A). BLASTn analysis identified TuMV as its closest member but only with 12% genome coverage. Further BLAST with the encoded protein showed MMV shared highest aa sequence identity (57%) with the tentative potyvirus PKV1 with 90% sequence coverage, indicating that MMV was potentially a new potyvirus. Sequence alignment with other potyviruses and BLASTp analyses revealed nine putative proteinase cleavage sites: _391_Y/S_392_, _849_G/G_850_, _1198_Q/A_1199_, _1251_Q/S_1252_, _1894_Q/S_1895_, _1947_Q/A_1948_, _2142_E/G_2143_, _2385_Q/S_2386_ and _2903_Q/N_2904_ for processing into the ten mature proteins (Figure 3A). The recently identified ORF encoding the PIPO protein [34] was identified from a G_2_A_7_ motif at position 3082–3090 within the P3 region protein in the +2 reading frame. The conserved motifs HX8DX31GXSG and RG in P1 were located at aa positions 299–343 and 366–367 [35]. In HC-Pro, the conserved motifs HXCX27CX2C [36], KITC, FRNK, IGN and PTK were found at aa positions 416–449, 443–446, 572–575, 641–643 and 701–703 respectively. In P3, the conserved residues EPY-(X)7-SP-(X)2-L were at aa position 882–896 of the polyprotein [37]. In RNA helicase CI, the conserved motifs GXXGXGKS (aa 1336–1343), VLLVEPTRPL (aa 1356–1365) [38], DECH (aa 1425–1428), VKVSATPP (aa 1451–1458), LIYV(aa 1503–1506), VATNIIENGVTL (aa 1554–1565) and GERIQRLGRVGR (aa 1598–1609) were all found. In NIa-Pro, the motif H(X)3T(X)2GHCG responsible for the proteinase activity [39], was identified at position 2284–2294. In NIb, the conserved residues SLKAEL and ADGSQFD were located at positions 2555–2560 and 2632–2638, respectively. The conserved amino acids FDSS at position 2637–2640 of the polyprotein were located 267 aa upstream of the putative NIb/CP cleavage site. The conserved motif (S/T)G-(X)3-T(X)3-N(S/T)(X)18-37GDD began at position 2695. In CP, the DAG motif, which interacts with PTK of HC-Pro to regulate potyviruses transmission by aphids [40], was located at position 2909–2911. The three consensus motifs found in the CP of potyviruses [41] were also found in MMV (MVWCIENGTSP, 3044–3054; AFDF, 3,127–3,130; QMKAAA, 3147–3152). Complete genome nt sequence analysis showed MMV shared highest sequence identity (57%) with some TuMV and plum pox virus (PPV) isolates, and the CP shared highest aa sequence identity (63.5%) with the CP of TuMV, which was below the threshold value used to discriminate between species and strains within the genus *Potyvirus* [42]. To further examine the relationship of MMV with other definite members in *Potyvirus*, polyprotein aa sequence based phylogenetic tree was constructed using the neighbor-joining (NJ) method implemented in the MEGA6.06 program and MMV clustered closest with PPV (Figure 3B).

### 3.5. Characterization of Small RNAs Derived from Marigold Mosaic Virus

**Figure 4:**
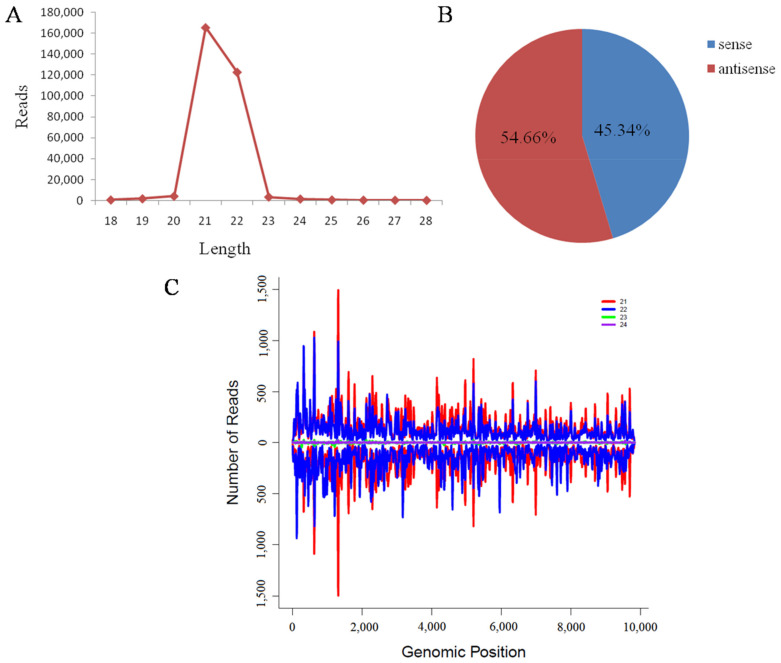
Profile of small RNAs derived from marigold mosaic virus (MMV). (**A**): Size distribution of 18–28 nt siRNAs derived from MMV. (**B**): the polarity distribution of MMV derived siRNAs. (**C**): The single-nucleotide resolution map of vsiRNAs along MMV genome, in either sense (bars above the axis) or antisense (bars below the axis) strand.

After small RNA alignment with the MMV genome as query, a total of 300,965 small RNAs with the length of 18–28 nt derived from MMV were gained and 21- (165,393) and 22-nt (122,794) reads were the dominant (Figure 4A), accounting for 54.9% and 40.8% respectively, which was in accordance with other reports [43,44]. To explore the origin of the vsiRNAs derived from MMV, their polarity distribution was further characterized. There was a slight bias towards MMV siRNAs derived from the antisense strand (164,519, 54.66%) of the viral RNA (Figure 4B). Single-nucleotide resolution maps of 21–24 nt small RNAs along the MMV genome showed that these small RNAs distributed continuously, but heterogeneously along the genome (Figure 4C), and there were strong hotspots in the 5′ terminal located in the untranslated region, P1 and HC-Pro region.

### 3.6. Field Distribution of Candidate Viruses

To explore the prevalence of the five candidate viruses in marigold plants field samples were collected and RT-PCR was performed with a specific primer pair for each virus (Table S1). CMV was detected in all the 24 symptomatic samples collected from Huairou and Yanqing (Table 2), indicating the wide distribution of CMV in marigold. Five plants from Huairou were MMV positive and this virus was not detected in the samples from Yanqing (Table 2). MCV was detected in three of the symptomatic and two of the asymptomatic plants from Huairou (Table 2), indicating the cryptic nature of this virus. TuMV was detected in twelve of the symptomatic plants (12/15) from Huairou and seven (7/9) from Yanqing (Table 2). For BBWV-2, only 6 plants (6/15) from Huairou were detected (Table 2).

## 4. Discussion

Since its first description demonstrating that the viral siRNAs could be used for viral genome assembly and virus discovery, sRNA deep sequencing has been widely used in virus identification and a large number of new viruses were recorded [45,46]. But the small size and discontinuity of the siRNA reads and the low number of reads may complicate the genome assembling process, even for accurate virus detection [47,48]. Recently studies reported that deep sequencing of the ribo-depleted total RNA outperformed the sRNA data in terms of the percentage of coverage that could be obtained particularly with the de novo assembled contigs [49]. In this study, sRNA was firstly conducted to identify the potential viruses associated with marigold mosaic disease and the viruses CMV, BBWV-2, MCV and MMV were identified, but no contigs from MCV dsRNA2 and TuMV were gained. In the ribo-depleted RNA sequencing, all the genomic sequences covering nearly the complete genome were gained, which was demonstrated to be more effective in virus identification and genome sequence characterization. This may be associated with the sequencing depths or low virus titer.

CMV is the type member of the genus *Cucumovirus* in the family *Bromoviridae*, and infects more than 1200 plant species with worldwide distribution. Based on the serology and sequence characterization, CMV strains have been classified into three subgroups, subgroup IA, IB and II [50]. In this study, the CMV isolate infecting marigold share high nt sequence identity with the isolates SL, WSJ and Tsh, which are all in group II. Further field survey and CP sequence analysis show that CMV of group II strains are widely distributed in marigold plants in Beijing. TuMV in the *Potyvirus* of *Potyviridae* is known to infect at least 318 species in over 43 dicot families with worldwide distribution. Based on host type, TuMV is grouped into two major groups: [B]-host-type infecting species of *Brassica* but not *Raphanus sativus* and [BR]-host-type infecting both *Brassica* and *Raphanus* [51]. Further phylogenetic groups of the basal-B, basal-BR, Asian-BR, and world-B lineages identified based on the complete genome sequence analysis [52]. Sequence comparison shows TuMV-marigold shares highest nt sequence identity with the isolates HRD, CHSE1AB, CHAJ23 and CHN342 in the Asian-BR subgroup, indicating that TuMV-marigold is a Asian-BR subgroup member. However, the pathogenicity and host range need to be further clarified. BBWV-2 in the *Fabavirus* of *Secoviridae* is an important virus, infecting both crop and ornamental species with worldwide distribution. Recently in China BBWV-2 has been reported in many new host plants and here BBWV-2 is firstly reported in marigold, which enriches our knowledge of its host range.

The genus *Potyvirus* is the largest group of the plant positive-strand RNA viruses, containing 183 currently approved species [42]. In this study, a new potyvirus tentatively named MMV is reported and its genome characterization is described. Conserved features are identified in MCV encoded proteins, excepted for the NIb/CP cleavage site of Q/N, which is less common and only reported in tobacco vein banding mosaic virus [53]. Compared with many other plant viruses, potyviruses have relatively narrow host ranges and are primarily transmitted by aphids in a nonpersistent manner. The conserved motifs KITC, PTK in HC-Pro and DAG in CP have been demonstrated to play vital roles in aphid transmission of potyviruses [54], and these motifs are also present in MMV, thus MMV is likely to be also aphid transmitted, but the detailed aphid species should be further examined. Recombination is an important role in potyvirus evolution but no evidence of recombination was detected in MMV using the RDP4 software conducted with other potyviruses. Plant viruses also evolved by adapting to one or more new hosts, and thus the host range of MMV should be further characterized.

Viruses in the family *Partitiviridae* have been shown to infect plants, fungi, and protozoa, and phylogenetic analysis based on amino acid sequences of RdRPs shows all the members cluster into five clades corresponding to the five partitivirus genera, which could be also characterized with the hosts and genome segment and protein lengths [55]. With the advent of next generation sequencing, many new partitiviruses have been identified in plants, fungi and even insects [56,57]. In this study, a new partitivirus infecting marigold named MCV was discovered and phylogenetic analysis shows that MCV is a member of the genus *Deltapartitivirus*. Conserved genomic features in delta-partitiviruses are also found in MCV. Partitiviruses consistently mediate persistent infections of their hosts and are classically considered to have few, if any, deleterious effects on host cells and are usually asymptomatic in plants. Moreover, some plant partitivirus genes appear to be associated with mutualistic host effects [58,59], but there are exceptions. For example, the cannabis cryptic virus was the solevirus in hemp plants with streaking [60], but there was no specific correlation between the observed symptom and the detected virus. Recently new putative insect infecting partitiviruses such as osugoroshi viruses have been discovered, and one is thought to be associated with late male-killing in the *Homona magnanima* population [59]. In the field survey, MCV is also present in asymptomatic marigold plants, and thus the role of MCV in the marigold-virus interaction system needs to be further explored.

Small RNA analysis shows that vsiRNAs derived from the MMV genome are mainly 21- and 22-nt in length (Figure 4A), suggesting that the marigold homologues of DCL4 and DCL2 may be the predominant Dicer-like ribonucleases involved in MMV vsiRNA biogenesis [61]. Polarity distribution analysis reveals approximately equal ratios of sense and antisense vsiRNAs (Figure 4B), indicating that most vsiRNAs would be produced from the replicative intermediate as dsRNA precursors comprised of sense and antisense strands. However, hotspots of sense and antisense strand are also observed in the single-nucleotide resolution map (Figure 4C), and the mechanism involved in the uneven distribution of vsiRNAs along the virus genome needs to be further investigated. VsiRNAs not only target the viral genome for degradation, but also could target host transcripts at post-transcriptional level [62], and the function of the vsiRNAs derived from MMV in virus-plant interaction still needs for exploration.

## 5. Conclusions

In summary, mixed infection of five viruses infecting marigold was identified by deep sequencing, including TuMV and BBWV-2, first reported in marigold, and two new viruses, marigold mosaic virus in the *Potyvirus* and marigold cryptic virus in the *Deltapartitivirus*. This work expanded the current knowledgeof the virome of marigold. However, the association between the observed symptoms and these viruses should be further investigated.

## Figures and Tables

**Table 1:** Related viruses identified in the deep sequencing data by BLAST analysis.

Candidate Viruses		sRNA Sequencing			RNA-Seq		
		Number of Contigs	Assembled Length	Genome Coverage	Number of Contigs	Assembled Length	Genome Coverage
broad bean wilt virus 2		84	8,577	-	5	9,465	-
	RNA1	53	5,848	98.2%	3	5,926	99.5%
	RNA2	31	2,729	76.0%	2	3,539	98.6%
cucumber mosaic virus		34	6,821	-	4	8,634	-
	RNA1	9	2,518	74.2%	1	3,390	99.9%
	RNA2	10	2,461	80.8%	1	3,040	99.9%
	RNA3	15	1,842	83.5%	2	2,204	99.9%
turnip mosaic virus		-	-	-	1	9,811	100%
polygonatumkingianum virus 1		27	8,247	87.8%	1	9,798	100%
ambrosia cryptic virus 2		6	454	-	2	3,042	-
	dsRNA1	6	454	28.2%	1	1,583	98.4%
	dsRNA2	-	-	-	1	1,459	85.8%

**Table 2:** Candidate virus detection in the field.

Candidate Virus	Samples from Huairou		Samples from Yanqing	
	Symptomatic	Asymptomatic	Symptomatic	Asymptomatic
CMV	15/15	0/6	9/9	0/4
TuMV	12/15	0/6	7/9	0/4
BBWV-2	6/15	0/6	0/9	0/4
MMV	5/15	0/6	0/9	0/4
MCV	3/15	2/6	0/9	0/4

## Data Availability

The high through-put sequencing data was submitted to GenBank (PRJNA716133) and the genome sequences were also available in GenBank. Materials described in the manuscript, including all relevant raw data, are also freely available to any scientist wishing to use them for non-commercial purposes upon request via e-mail with the corresponding author.

## Supplementary Materials

The following are available online at https://www.mdpi.com/article/10.3390/v13071254/s1, Table S1: Primers used in this study.
