# Supplementary material for: Metagenomic Analysis of Marigold: Mixed Infection Including Two New Viruses
Source: Viruses. 2021 Jun 28;13(7):1254. doi: 10.3390/v13071254 (PMC8310094; doi:10.3390/v13071254)
Supplement: Supplementary file 1 [file viruses-13-01254-s001.zip › viruses-1163809-supplementary.pdf]

Table S1 Primers used in this study

Virus	Forward primer	Reverse primer
Primer pair used for virus identification		
**BBWV 2**	ACCCGTACGC AACAGTTATG GCAGG	GAT CTTTCTGAAT TGTCTCGGTT TGG
**TuMV**	CGTTTT CAAAGCAACC AACAACAACG	AACTCAATGT TCGCACTCTT CGCCTT
CMV	GCCATCAAGA AGATGGCTAC TGAGTGT	GTTACAACTC ACACGGGACT CTAGACT
**MMV**	GATT GTGGAGAAGT CGCCGGTATT GT	TCACGCACCA TCTTCGTAAA CTCTT
**MCV**	GGACGACAGTCCGAAACTATCTATGAAG	CTCTGATGGA TCATAATACC TGTCATAGT
Primer pair used for complete genome sequencing		
TuMV1	CATACACAAAACGATTAAAGCAAACAC	TTTGCTTCAG CAGCTATTGT GACTAG
TuMV2	TAGTCGTAAA GTTTCAAGTA GTGTTTG	TTAGGTACTT CACTTAAGTC CATAGGCT
TuMV3	ACCACACATA CCACTAAAGG TATGTG	TGTCCCTTGC ATCCTATCAA ATGTTAAG
BBWV-2 RNA1-1	TATTAAAACA AACAGCTTTC GTTCCG	TCTCTGAATT ATCTCGATTA GGAATATC
BBWV-2 RNA1-2	CACTCCAATC TTTATGGTGTATGTCAG	ACTTGGGAAA AGGCATTTCA ACCCTG
BBWV-2 RNA2	ATTGAAACAA ACAGCTTTCG TTCCGAG	CTAAAGCATA TTTATGTAGG AAGCTTT
MMV1	AGTTGACCGT TATAATTTGA AGCATGG	GCCTCCCATATCTGCTGTACTTTCTG
MMV2	AGAGTTAACTTGGTTGGAAAAATTATCTG	TTCTGGAAAT CCTGCAATTG ATGCTG
MMV3	GATATCAACA TAGTTCAAGA ACACTTC	CATAGTGTGC AAATTTTGAT TAACAT
MCV dsRNA1	TACGGAAGAC TTACATTATG TCAGTCT	GAAG CAACTCCATA AATCCTTTTA AGCCT
MCV dsRNA2	TACACTAATG GCAGAGAAAC GTCCG	ATCAAAGTCG ATTACAAAAT TGTTGAAAC
CMV RNA1	AGAGCGTACG GTTCAACCCC TGCCT	GTCGAGTATG GTCTCCTTAT GGAGAAC
CMV RNA2	CTCAAGAGCG TATGGTTCAA CCCCTG	GTCGAGTATG GTCTCCTTAT GGAGAAC
CMVRNA3	ACCACTTTCTTTTCACGTCG TGTCGCGT	GTCGAGTATGGTCTCCTTAT GGAGAACCTG
Primer for RACE	5'RACE	3'RACE
**MMV**	GATTACGCCAAGCTTCTGCTTTCT TCGTGACGAA CTCGAACTGC	GATTACGCCAAGCTT GACGCCAGCA CGAGCTAAAG AGGCGC
MCV dsRNA1	GATTACGCCAAGCTT CTTAC AGCTTCGACG CTCTGTCGCC	GATTACGCCAAGCTT CCTGAGT ACCCCGTTGA AGACGGAAGT
MCV dsRNA2	GATTACGCCAAGCTT TGC CGGTGGGGAG AGGGAACTTC TCG	GATTACGCCAAGCTT GATGATGC TGAATCGTCC GAAGCTCCAG
BBWV RNA1	GATTACGCCAAGCTT CT CCTCTTTTTC CTCCATAAAT TCACAGG	GATTACGCCAAGCTT CTTCTGAATC GAGCATCCTT TCCTTATG
BBWV RNA2	GATTACGCCAAGCTTGAAGGGCGCAACAA TATATGCAAA TTCGAG	GATTACGCCAAGCTT GGCTCT AACGTCGAAT TTGCTGGCC
